# Supplementary material for: AQoLS-Brief: Development and psychometric properties of a short version of the alcohol quality of life scale
Source: PLoS One. 2025 Apr 29;20(4):e0321365. doi: 10.1371/journal.pone.0321365 (PMC12040188; doi:10.1371/journal.pone.0321365)
Supplement: Attachment — Submitted filename: PLOS ONE review_12.2024.docx [file pone.0321365.s001.docx]

Thank you for the opportunity to review this manuscript. The current study creates and validates a brief version of the Alcohol Quality of Life Scale (AQoLS-Brief) in a sample of recently detoxified patients with alcohol use disorder. Please find me critiques below.

- The external validity variables, besides the MoCA, were very focused on alcohol use-related constructs. The variables included are good, but some additional variables not related to alcohol specifically but that map onto some of the subscales or global quality of life would strengthen the convergent and discriminant validity analysis (Clark & Watson, 2019). Candidate variables could include scales of global quality of life, depression, anxiety, physical health pr medical history, relationship functioning, socioeconomic status, sleep quality, and so on. If none of these variables were measured in the original data collection, then at the very least the lack of these measures should be included as a limitation.
  - Clark, L. A., & Watson, D. (2019). Constructing validity: New developments in creating objective measuring instruments. Psychological Assessment, 31(12), 1412–1427. <https://doi.org/10.1037/pas0000626>
- Related to the point above, it would be useful to examine validity predictors in a multiple regression together (as opposed to bivariate correlations only). This would allow for parsing apart which constructs are most strongly/uniquely associated with the AQoLS-Brief.
- I am confused why it was hypothesized that there would be no correlation between the AQoLS-Brief and cognitive impairment. Cognitive impairment is a well-established correlate of alcohol use disorder (Bernardin et al., 2014; Wang et al., 2023), even after recovery (Stavro et al., 2013). The current manuscript even cited an article demonstrating the utility of the MoCA as a screening tool for alcohol-dependent individuals, and the data comes from a clinical trial in which patients undergo a cognitive training program. There is also evidence that cognitive impairment and quality of life are negatively associated (Hill et al., 2017). What is the rationale that alcohol-related impairment in life would not be associated with cognitive impairment?
  - Bernardin, F., Maheut-Bosser, A., & Paille, F. (2014). Cognitive impairments in alcohol-dependent subjects. *Frontiers in psychiatry*, *5*, 78.
  - Hill, N. L., McDermott, C., Mogle, J., Munoz, E., DePasquale, N., Wion, R., & Whitaker, E. (2017). Subjective cognitive impairment and quality of life: a systematic review. *International psychogeriatrics*, *29*(12), 1965-1977.
  - Stavro, K., Pelletier, J., & Potvin, S. (2013). Widespread and sustained cognitive deficits in alcoholism: a meta‐analysis. *Addiction biology*, *18*(2), 203-213.
  - Wang, G., Li, D. Y., Vance, D. E., & Li, W. (2023). Alcohol use disorder as a risk factor for cognitive impairment. *Journal of Alzheimer's Disease*, *94*(3), 899-907.
- I have some reservations about the decision to exclude factorial analysis and believe that a confirmatory factor analysis (CFA) should be estimated. I understand trying to retain the original 7-dimension structure, but surely the intention of this scale is to be used as a screen for general alcohol-related quality of life impairment, suggesting the measure will be treated as a single factor scale. A CFA would inform how the measure performs as a single, unidimensional factor of alcohol-related quality of life impairment (Briggs & Cheek, 1986; Simms, 2008). Examining factor loading strength and patterns of the brief dimension could even be used to guide item selection. Currently as it stands, the two selection criteria tell us whether the items selected are representative of their original scale? A CFA would tell us do these selected items hang together as a single construct that can be used meaningfully? Moreover, the original studies examining psychometric performance of the AQoLS reported: “The preliminary principal component analysis indicated a substantive principal dimension accounting for 42 % of the variance, thus indicating the appropriateness of a total AQoLS score summing the items” (Luquiens et al., 2016). While a 7-factor solution was ultimately settled on, it seems that testing a single factor CFA is appropriate given some of the preliminary work with the full measure and the purpose of using brief measures more broadly.
  - Briggs SR, & Cheek JM (1986). The role of factor analysis in the development and evaluation of personality scales. Journal of Personality, 54, 106–148.> doi: 10.1111/j.1467-6494.1986.tb00391
  - Luquiens, A., Whalley, D., Laramée, P., Falissard, B., Kostogianni, N., Rehm, J., ... & Aubin, H. J. (2016). Validation of a new patient-reported outcome instrument of health-related quality of life specific to patients with alcohol use disorder: the Alcohol Quality of Life Scale (AQoLS). Quality of Life Research, 25, 1549-1560.
  - Simms, L. J. (2008). Classical and Modern Methods of Psychological Scale Construction. Social and Personality Psychology Compass, 2(1), 414–433. https://doi.org/10.1111/j.1751-9004.2007.00044.x
- I believe the effect of the sample and assessment time periods on validity associations needs to be discussed more in the manuscript. For example, in the discussion, it is stated that the low correlation between the number of HDD in the last 4 weeks and the AQoLS-brief suggests that drinking intensity has limitations as an outcome. While there is some truth to this statement, I think the association between the number of HDD in the last 4 weeks and the AQoLS-brief needs some more discussion. If I am understanding the inclusion criteria correctly, patients recently detoxified and, at minimum, did not drink in the last 7 days (up to 30). This means that heavy drinking for individuals was at minimum ¼ lower, and potentially completely absent, in the assessment period. Consequently, this inclusion criteria artificially attenuates the association between drinking intense and quality of life. In my opinion, and if I am understanding things correctly, this is likely the best explanation for why heavy drinking and quality of life from drinking were not significantly associated with one another. The conclusion above about this association should be tempered.
  - The association with the CGI-severity score has a similar issue. It is concluded that assessing impairment in an inpatient setting does not capture the full spectrum of quality-of-life alcohol impairment. Again, I do not inherently disagree with this statement, but the past 7 days, by definition of the inclusion criteria, means that there was not alcohol use present during the time of the CGI-severity assessment. Would this not artificially decrease the disease severity score to be lower and thus attenuate the CGI-severity and AQoLS-brief association?
- I would like to hear more information about excluding patients currently experiencing withdrawal symptoms from the current analysis. What proportion of the sample does this exclude? Does this only include extreme, life-threatening withdrawal symptoms, or even more mild withdrawal symptoms? If it is life-threatening symptoms only then that makes sense for safety purposes. However, exclusion based on mild withdrawal symptoms may bias the sample unnecessarily.
- I am wondering why other item selection analyses were not used to guide item selection, such as item discrimination and item difficulty. Some rationale as to why these were not used would be helpful.
- This is a minor, but there are so many acronyms it is difficult to follow the manuscript at times. I recommend only using very common acronyms (e.g., AUD, DSM-5) and writing out most other acronyms.
- This is also minor, but please proofread the manuscript for correct grammar. For example, on lines 52-54, it reads: “However, its length could limit its use in clinical trials, where subjective measurements are not often the primary judgment outcome (Luquiens et al. 2012) and there is often numerous and time spending measures.” Instead of “there is often numerous and time spending measures,” I assume the authors mean, “there is often numerous and time intensive measures.” There are several places throughout the manuscript where the wording or grammar is slightly off.
