# Supplementary material for: AQoLS-Brief: Development and psychometric properties of a short version of the alcohol quality of life scale
Source: PLoS One. 2025 Apr 29;20(4):e0321365. doi: 10.1371/journal.pone.0321365 (PMC12040188; doi:10.1371/journal.pone.0321365)
Supplement: Attachment — Submitted filename: PLOS ONE review_1_210125.docx [file pone.0321365.s003.docx]

We would like to thank the reviewers for their thoughtful comments and suggestions, which have helped improve the quality of our manuscript. Below, we provide our detailed responses to each of the comments, outlining the revisions made and clarifications provided to address the points raised.

Reviewer #1: Introduction
1. Line 43: Add a concise explanation of the COSMIN criteria and highlight their relevance.

R1. We reformulated the 1^st^ paragraph to explain the COSMIN criteria and highlight their relevance: P4L41“*The Alcohol Quality of Life Scale (AQoLS) is a patient-reported outcome measure (PROM) developed with significant input from patients with current or remitted alcohol use disorder (AUD)* (Luquiens et al. 2015)*, in accordance with COSMIN (COnsensus-based Standards for the selection of health Measurement INstruments) guidelines* *(Mokkink et al. 2018). These guidelines emphasize ensuring strong content validity by involving patients in identifying relevant elements through concept elicitation and conducting cognitive interviews to assess the comprehensiveness, comprehensibility, and relevance of the items in alignment with the intended construct”*

2. Line 43: While the Alcohol Quality of Life Scale (AQoLS) is described as having excellent internal consistency, other psychometric properties (e.g., test-retest reliability, validity in different populations) of both the original and brief versions are not addressed. Adding this would make the justification for the AQoLS more robust.

R2. We added the following sentence to provide additional information on the psychometrics of the AQoLS: P4L589“*The AQoLS total score showed an intraclass correlation coefficient at 2 weeks in stable out-patients with alcohol use disorder of 0.80 (Higuchi, Moriguchi, and Tan 2020)*. “As the Brief version was not developed, we cannot provide further psychometrics of the AQoLS-Brief in other populations.

3. Line 54: ‘A shorter version would be more convenient for both researchers and users.’ -- What was the rationale for using the shorter version of AQoLS-Brief? It is not clear. Clearly state the research gap this study aims to fill.

R3. We added the following explanation in the introduction to clarify the rationale for using a shorter version of the AQoLS:: P4L63“*Using a shorter version of a scale is more convenient as it reduces the time burden on both participants and researchers, making it easier to administer in clinical or research settings where time constraints are often present. A shorter scale also improves participant engagement and completion rates, while still retaining the key measurement properties and relevance of the longer version. This helps ensure more efficient data collection without compromising the quality of the information gathered.*“

4. Provide more specifics on how the AQoLS-Brief will facilitate discrete choice experiments. Will it reduce complexity or address specific methodological challenges? This clarification will underline the importance of this study.

R4. We provided more details: P5L87 “*Short versions of scales are needed for a Discrete Choice Experiment due to the complexity of constructing pairwise combinations. These combinations involve comparing multiple health states defined by different attributes, and as the number of attributes and levels increases, the total number of possible pairwise combinations grows exponentially. By using condensed versions of scales, which reduces the number of attributes and levels, the number of combinations is significantly reduced, making the experimental design more manageable. This simplification helps avoid participant fatigue and ensures the choice tasks are feasible and understandable, leading to more reliable data for deriving utility values*.”

Study Design and Methods
5. Line 81: Write the full abbreviation of TRAIN study.

R5. We detailed P6L110“TRAIN study (TRAining INhibition in alcohol use disorder)”

6. Line 97: ‘Patient had to be aged 18 to 65 with current AUD, according to the DSM-5, classed at least as high drinking risk level (men: alcohol consumption >60 g/day; women >40 g/day) as regarding the alcohol consumption in the last 4-week drinking period.’ -- break it into two lines.

R6. We broke it into two lines.

7. Line 100: Why these specific criteria (e.g., 7-30 days abstinence) were selected. Does this enhance the reliability of baseline data, reduce variability, or align with clinical practice?

R7. These criteria were necessary as the intervention aimed to prevent relapse in individuals with alcohol use disorder who had recently undergone detoxification. They were also required to enhance the reliability of baseline data and to minimize recall bias related to describing the last drinking period.

8. Please note that the inclusion criteria started from lines 97 – 103. Line 100: include ‘also’ – Inclusion criteria also included no benzodiazepines use for at least 3 days prior to inclusion, to avoid interference with alcohol intoxication or withdrawal medication on the neuropsychological assessments, and any recall bias on drinking.

R8. We included “also”.

9. Consider adding more discussion on how the AQoLS-Brief was developed with input from patients and how this ensures its relevance.

R9. We modified a couple of paragraphs in the introduction and methods: P4L41“*The Alcohol Quality of Life Scale (AQoLS) is a patient-reported outcome measure (PROM) developed with significant input from patients with current or remitted alcohol use disorder (AUD)”. P8L193 “We selected one item per dimension using a priori rules demonstrating the predominant nature of the item within each dimension. This choice was made to respect the input of patients at the time of the development of the AQoLS, that lead in a qualitative analysis to the identification of the 7 dimensions* (Luquiens et al. 2015) *confirmed in the later validation study (Luquiens et al. 2016).”*

10. Line 171: Write the full abbreviation of MoCA score.

R10. We corrected for: “Montreal Cognitive Assessment (MoCA)”

Discussion
11. The discussion could better connect the results to the study’s objectives and implications for future research or clinical practice. Discuss how the AQoLS-Brief improves on the original AQoLS in terms of usability and application. Address potential limitations of the study (e.g., sample representativeness, reliance on specific statistical rules for item selection) and suggest how they could be addressed in future research.

R11. We modified the discussion: P19L339 “*The AQoLS-Brief, a 7-item short version of the AQoLS, has demonstrated good internal consistency and unidimensionality, supporting the calculation of a total score. It is a valid and user-friendly measure of the impact of alcohol on quality of life, as perceived by individuals with lived experience. By offering a more concise alternative, the AQoLS-Brief could alleviate barriers associated with the use of the longer AQoLS version, making it more practical for clinical and research settings. Furthermore, it meets the necessary standards for use in a future discrete choice experiment to develop a specific health utility score.”*

We added several limits in the discussion: P20L387*“Our study has certain limitations, as we chose not to conduct a new exploratory factor analysis, focusing instead on a confirmatory approach. This decision was made to retain the previously described seven-dimension structure, which reflects patients’ perspectives, by selecting the best item from each dimension. The a priori rules for selecting these items were a posteriori validated* *by the additional analyses, which demonstrated the relevance of the selected items and the strong psychometric properties of the 7-item AQoLS-Brief scale.* *We included a limited number of external variables to assess divergent and convergent validity. Notably, we did not measure quality of life using another instrument, as the data were collected as part of a randomized clinical trial, and adding another measure would have been too time-consuming*.*”*

Reviewer #2:

1. The external validity variables, besides the MoCA, were very focused on alcohol use-related constructs. The variables included are good, but some additional variables not related to alcohol specifically but that map onto some of the subscales or global quality of life would strengthen the convergent and discriminant validity analysis (Clark & Watson, 2019). Candidate variables could include scales of global quality of life, depression, anxiety, physical health pr medical history, relationship functioning, socioeconomic status, sleep quality, and so on. If none of these variables were measured in the original data collection, then at the very least the lack of these measures should be included as a limitation.
   - Clark, L. A., & Watson, D. (2019). Constructing validity: New developments in creating objective measuring instruments. Psychological Assessment, 31(12), 1412–1427. <https://doi.org/10.1037/pas0000626>

R1. We thank the reviewer for his/her request and suggestions for further analysis of external validity analyses.

We added psychological variables in the manuscript that were not reported before that can reflect anxiety and depression:

*Methods: P8L181 “Psychological variables - We collected the Affective Neuroscience Personality Scales (ANPS) and the Substance Use Risk Profile Scale (SURPS) to assess personality traits. The ANPS measures six primary emotional traits: seeking, care, play, fear, anger, and sadness. It was initially validated by Davis and Panksepp (Davis, Panksepp, and Normansell 2003) providing a framework to explore the neurobiological basis of personality. The SURPS assesses four personality dimensions associated with substance use vulnerability: anxiety sensitivity, hopelessness, impulsivity, and sensation seeking, with its initial validation conducted by Woicik et al. (Woicik et al. 2009). Both scales are psychometrically robust and widely used in research to link personality traits to behavioral and emotional outcomes.“*

*P10L236 “We hypothesized a moderate correlation […] with dimensions of the psychological scales ANPS and SURPS related to anxiety-depression, i.e. ANPS sadness and fear dimensions, and SURPS anxiety sensitivity and hopelessness dimensions*.”

P16L319 AND 323 *“Results:*

“*We found a moderate positive correlation […] with sadness dimension of the ANPS and the dimensions hopelessness and anxiety sensitivity of the SURPS*.”

|  | AQoLS-Total | AQoLS-Brief |
| --- | --- | --- |
| Age (years) | -0.16 [-0.29; -0.03] | -0.19 [-0.32; -0.06] |
| CGI-severity | 0.03 [-0.11; 0.17] | 0.04 [-0.09; 0.18] |
| Moca Score | -0.03 [-0.16; 0.11] | -0.06 [-0.20; 0.07] |
| Number of DSM alcohol criteria | 0.40 [0.28; 0.51] | 0.40 [0.28; 0.51] |
| Length since the onset of alcohol use disorder (years) | 0.22 [0.08; 0.34] | 0.20 [0.07; 0.32] |
| Number of heavy drinking days (28d) | 0.10 [-0.04; 0.23] | 0.08 [-0.05; 0.22] |
| ANPS score |  |  |
| Fear | 0.19 [0.06; 0.32] | 0.16 [0.03; 0.29] |
| Sadness | 0.41 [0.29; 0.51] | 0.36 [0.23; 0.47] |
| SURPS score |  |  |
| Anxiety Sensitivity | 0.28 [0.16; 0.40] | 0.26 [0.13; 0.38] |
| Hopelessness | 0.32 [0.19; 0.43] | 0.29 [0.16; 0.41] |

*Discussion: P19L359 and 366 “We found a moderate correlation with DSM-5 criteria for AUD, the sadness dimension of the ANPS and the dimensions hopelessness and anxiety sensitivity of the SURPS. ““The moderate to low correlations between anxiety- and depression-related dimensions of the ANPS and SURPS were also expected. This aligns with findings that anxiety- and depression-related items in non-specific HRQOL instruments are often the only dimensions affected by alcohol, whereas other dimensions remain unaffected (Luquiens et al. 2012). Consequently, such non-specific instruments fail to capture the full spectrum of alcohol's impact on HRQOL, supporting the use of specific tools, such as the AQoLS, which are designed to comprehensively assess the unique impact of alcohol on quality of life”*

*We also added the description of the 2 scales in table 1. P11*

| Variable | N=227 |
| --- | --- |
| ANPS total score (mean, sd) | 63.3 (9.7) |
| Sadness | 10.2 (3.3) |
| Fear | 10.1 (3.7) |
| Care | 12.8 (3.1) |
| Anger | 7.4 (3.5) |
| Play | 10.9 (3.3) |
| Seeking | 11.9 (3.1) |
| SURPS Total score (mean, sd) | 56.1 (8.0) |
| Hopelessness | 16.7 (4.3) |
| Anxiety Sensitivity | 15.2 (2.9) |
| Sensation Seeking | 13.0 (3.4) |
| Impulsivity | 11.4 (3.0) |

Moreover, we added in the limit section the following limit: P21L400 “*We included a limited number of external variables to assess divergent and convergent validity. Notably, we did not measure quality of life using another instrument, as the data were collected as part of a randomized clinical trial, and adding another measure would have been too time-consuming.”*

2. Related to the point above, it would be useful to examine validity predictors in a multiple regression together (as opposed to bivariate correlations only). This would allow for parsing apart which constructs are most strongly/uniquely associated with the AQoLS-Brief.

R2. As suggested by the reviewer, we provided a multiple linear regression between AQoLS-brief and the external variables:

Methods:

P10L243*” We performed a multiple linear regression explaining the AQoLS-brief/AQoLS by the external variables.”*

Results: P18L328 *“Table 6 provides a multiple linear regression between AQoLS-brief and external variables.* *Severity of alcohol use disorder, as measured by the number of DSM-5 alcohol criteria, the HDD and the Dimension Sadness (ANPS) were the heaviest contributors to the AQoLS-Brief score. A younger age and length since the onset of alcohol use disorder also contributed significantly to the AQoLS -Brief score*. “

*Table 6: Multiple linear regression between AQoLS-Total /AQoLS-Brief and external variables*

|  | **AQoLS-Total  Coef [CI95%]** | **AQoLS-Brief  Coef [CI95%]** | **Variance Inflation Factor^a^ [CI95%]** |
| --- | --- | --- | --- |
| (Intercept) | -0.26** [-0.51; -0.02] | -0.27** [-0.52; -0.02] |  |
| Age (years) | -0.21*** [-0.33; -0.10] | -0.24*** [-0.35; -0.12] | 1.16 [1.06; 1.44] |
| Number of DSM alcohol criteria | 0.33*** [0.22; 0.44] | 0.33*** [0.22; 0.45] | 1.12 [1.03; 1.43] |
| Length since the onset of alcohol use disorder (years) | 0.22*** [0.11; 0.33] | 0.21*** [0.09; 0.32] | 1.14 [1.05; 1.43] |
| Dimension Fear (ANPS) | -0.02 [-0.14; 0.11] | -0.03 [-0.16; 0.09] | 1.36 [1.20; 1.65] |
| Dimension Sadness (ANPS) | 0.31*** [0.17; 0.45] | 0.27*** [0.13; 0.41] | 1.72 [1.47; 2.09] |
| Dimension Anxiety Sensitivity (SURPS) | 0.05 [-0.07; 0.17] | 0.07 [-0.05; 0.19] | 1.29 [1.15; 1.57] |
| Dimension Hopelessness (SURPS) | 0.09 [-0.03; 0.22] | 0.09 [-0.04; 0.21] | 1.42 [1.24; 1.72] |
| **Number of heavy drinking days** |  |  | 1.08 [1.01; 1.48] |
| 1-12 days | Ref | Ref |  |
| 13-27 days | 0.49*** [0.15; 0.84] | 0.50*** [0.15; 0.86] |  |
| 28 days | 0.28* [-0.01; 0.56] | 0.28* [-0.01; 0.56] |  |
| N | 224 | 224 |  |
| R2 | 0.40 | 0.38 |  |
| a: check for multicollinearity among independant variables. Should be less than 5 | | | |
| Levels of statistical significance: * p<0.10, ** p<0.05, *** p<0.01 | | | |
| Dependant variables and continuous independant variables are standardized to facilitate comparison of estimates | | | |

Methods were amended in accordance: P9L243 *“We performed a multiple linear regression explaining the AQoLS-brief/AQoLS by the external variables.”*

3. I am confused why it was hypothesized that there would be no correlation between the AQoLS-Brief and cognitive impairment. Cognitive impairment is a well-established correlate of alcohol use disorder (Bernardin et al., 2014; Wang et al., 2023), even after recovery (Stavro et al., 2013). The current manuscript even cited an article demonstrating the utility of the MoCA as a screening tool for alcohol-dependent individuals, and the data comes from a clinical trial in which patients undergo a cognitive training program. There is also evidence that cognitive impairment and quality of life are negatively associated (Hill et al., 2017). What is the rationale that alcohol-related impairment in life would not be associated with cognitive impairment?
   - Bernardin, F., Maheut-Bosser, A., & Paille, F. (2014). Cognitive impairments in alcohol-dependent subjects. *Frontiers in psychiatry*, *5*, 78.
   - Hill, N. L., McDermott, C., Mogle, J., Munoz, E., DePasquale, N., Wion, R., & Whitaker, E. (2017). Subjective cognitive impairment and quality of life: a systematic review. *International psychogeriatrics*, *29*(12), 1965-1977.
   - Stavro, K., Pelletier, J., & Potvin, S. (2013). Widespread and sustained cognitive deficits in alcoholism: a meta‐analysis. *Addiction biology*, *18*(2), 203-213.
   - Wang, G., Li, D. Y., Vance, D. E., & Li, W. (2023). Alcohol use disorder as a risk factor for cognitive impairment. *Journal of Alzheimer's Disease*, *94*(3), 899-907.

R3. We respectfully wish to offer a different perspective on the reviewer’s reasoning. We fully agree that “cognitive impairment is a well-established correlate of alcohol use disorder” and that, in other contexts, such as dementia or severe cognitive decline in the elderly, “cognitive impairment and quality of life are negatively associated.” However, despite carefully reviewing the references provided and conducting additional searches on PubMed (e.g., ["quality of life" "Montreal Cognitive Assessment" alcohol], ["quality of life" "cognitive impairment" "alcohol use disorder"]), we were unable to locate original data specifically supporting the suggestion that alcohol-related impairment in quality of life is associated with cognitive impairment.

The suggestion implies that individuals with no cognitive impairment would systematically experience a lower subjective impact of alcohol on their quality of life compared to those with severe cognitive impairment. Based on our clinical experience, this does not appear to be the case. Rather, the impact of alcohol on quality of life seems to depend more significantly on the severity of dependence and the duration of the disorder. We formulated our hypotheses accordingly.

To further substantiate our results, we explored the association between AQoLS-Brief scores and MoCA scores, categorized as <26 or ≥26 to indicate the presence or absence of severe cognitive impairment. The results are presented below:

| **Characteristics** | | **% (n)** | **Mean AQols (SD)** | **Mean AQols-Bref (SD)** |
| --- | --- | --- | --- | --- |
|  | **Moca Score** |  | p=0.865 | p=0.538 |
|  | <26 | 29.8% (67) | 48.9 (20.7) | 11.3 (5.0) |
|  | ≥26 | 70.2% (158) | 48.3 (20.0) | 10.9 (4.4) |

As shown, the AQoLS scores did not significantly differ between the two groups. Given that these additional analyses align with and further support the results already reported in the manuscript, we respectfully decided not to include them.

4. I have some reservations about the decision to exclude factorial analysis and believe that a confirmatory factor analysis (CFA) should be estimated. I understand trying to retain the original 7-dimension structure, but surely the intention of this scale is to be used as a screen for general alcohol-related quality of life impairment, suggesting the measure will be treated as a single factor scale. A CFA would inform how the measure performs as a single, unidimensional factor of alcohol-related quality of life impairment (Briggs & Cheek, 1986; Simms, 2008). Examining factor loading strength and patterns of the brief dimension could even be used to guide item selection. Currently as it stands, the two selection criteria tell us whether the items selected are representative of their original scale? A CFA would tell us do these selected items hang together as a single construct that can be used meaningfully? Moreover, the original studies examining psychometric performance of the AQoLS reported: “The preliminary principal component analysis indicated a substantive principal dimension accounting for 42 % of the variance, thus indicating the appropriateness of a total AQoLS score summing the items” (Luquiens et al., 2016). While a 7-factor solution was ultimately settled on, it seems that testing a single factor CFA is appropriate given some of the preliminary work with the full measure and the purpose of using brief measures more broadly.
   - Briggs SR, & Cheek JM (1986). The role of factor analysis in the development and evaluation of personality scales. Journal of Personality, 54, 106–148.> doi: 10.1111/j.1467-6494.1986.tb00391
   - Luquiens, A., Whalley, D., Laramée, P., Falissard, B., Kostogianni, N., Rehm, J., ... & Aubin, H. J. (2016). Validation of a new patient-reported outcome instrument of health-related quality of life specific to patients with alcohol use disorder: the Alcohol Quality of Life Scale (AQoLS). Quality of Life Research, 25, 1549-1560.
   - Simms, L. J. (2008). Classical and Modern Methods of Psychological Scale Construction. Social and Personality Psychology Compass, 2(1), 414–433. <https://doi.org/10.1111/j.1751-9004.2007.00044.x>

R4. We agree with the reviewer that it is interesting and performed a CFA:

Methods: P9L226*“We performed a confirmatory factor analysis (CFA) of the AQoLS-Brief to validate the unidimensionality of the construct being measured. We estimated the CFA model with the R lavaan package (Rosseel 2012) treating the seven items of AQoLS-Brief as ordinal variables (Gana and Broc 2018). It uses the diagonally weighted least squares method for the estimation (Jöreskog, Sörbom, and Inc 1989) encouraged when the sample is small and data violates normality .* *To assess the fit of the CFA model, the chi-square test, root mean square error of approximation, standardized root mean square residual, Tucker-Lewis Index, and Comparative Fit Index were examined. These measures were evaluated against established guidelines to determine the adequacy of the model fit.”*

Results: P16L300 “*Figure 1 presents the* Standardized loadings estimates of the confirmatory factor analysis *of the AQoLS-Brief.*

*Footnote : The overall model* $\chi^{2}$ *is 14.45 with 14 degrees of freedom. The p-value associated with this result is 0.417, which is non significant indicating that the observed covariance matrix matches the estimated covariance matrix within sampling variance.* *The value for* *root mean square error, an absolute fit index, is 0.012 i.e.below the 0.08 guideline. The 90 percent confidence interval for this root mean square error is [0.000; 0.066]. Therefore, even the upper bound of root mean square error is below the 0.08 guideline. We conclude that the root mean square error provides additional support for model fit. The standardized root mean square residual with a value of 0.045 is below the 0.08 guideline. Moving to the incremental fit indices, the Tucker-Lewis Index and Comparative Fit Index indices have both the value 1.000 which is greater than the guideline 0.94. In conclusion, the CFA results suggest that the AQoLS-Brief measurement model provides a good fit.*

Additionally, we calculated the Cronbach α and the average inter-item correlation for the AQoLS-Brief, the AQoLS and each of its dimensions to further document the relevance of the item selection. Average inter-item correlation for a set of items should be between 0.20 and 0.40, suggesting that while the items are reasonably homogenous, they do contain sufficiently unique variance so as to not be isomorphic with each other. For every item we also compute α-drop and average inter-item correlation--drop which are respectively define as Cronbach α excluding the particular item of the dimension and AIIC excluding the particular item of the dimension. The lowest the α-drop/ average inter-item correlation -drop for an item is, the more important this item is for this dimension.*”*

We added in the discussion: P19L340 “*It demonstrated […] unidimensionality, supporting the calculation of a total score.” P20L387“Our study has certain limitations, as we chose not to conduct a new exploratory factor analysis, focusing instead on a confirmatory approach. This decision was made to retain the previously described seven-dimension structure, which reflects patients’ perspectives, by selecting the best item from each dimension”*

5. a) I believe the effect of the sample and assessment time periods on validity associations needs to be discussed more in the manuscript. For example, in the discussion, it is stated that the low correlation between the number of HDD in the last 4 weeks and the AQoLS-brief suggests that drinking intensity has limitations as an outcome. While there is some truth to this statement, I think the association between the number of HDD in the last 4 weeks and the AQoLS-brief needs some more discussion. If I am understanding the inclusion criteria correctly, patients recently detoxified and, at minimum, did not drink in the last 7 days (up to 30). This means that heavy drinking for individuals was at minimum ¼ lower, and potentially completely absent, in the assessment period. Consequently, this inclusion criteria artificially attenuates the association between drinking intense and quality of life. In my opinion, and if I am understanding things correctly, this is likely the best explanation for why heavy drinking and quality of life from drinking were not significantly associated with one another. The conclusion above about this association should be tempered.

R5a.

We respectfully wish to clarify for the reviewer that, as outlined in the Methods section, we collected data on alcohol consumption during the 4-week period immediately preceding the last drinking occasion. To address any potential misunderstanding, we have reformulated the Methods section to make this point clearer: P8L176 *'We collected data on the time elapsed since the last drink and on alcohol consumption during the so-called 4-week drinking period leading up to the last drinking occasion.'*

This clarification ensures there is no impact or attenuation in the measurement of alcohol consumption intensity.

b) The association with the CGI-severity score has a similar issue. It is concluded that assessing impairment in an inpatient setting does not capture the full spectrum of quality-of-life alcohol impairment. Again, I do not inherently disagree with this statement, but the past 7 days, by definition of the inclusion criteria, means that there was not alcohol use present during the time of the CGI-severity assessment. Would this not artificially decrease the disease severity score to be lower and thus attenuate the CGI-severity and AQoLS-brief association?

R5b. We totally agree with the reviewer, and already pointed out this issue in the discussion: “*The very low level of the CGI-S with a 7-day recall period is a good illustration of the difficulty in assessing inpatients with alcohol use disorder, for whom the control of the stimuli could be a relief with a temporary suspension of symptoms associated with alcohol intoxication, particularly craving, and does not reflect the wide range of effects of alcohol on quality of life, as measured by the AQoLS and AQoLS-Brief. “*

We added this limit more explicitly in the discussion: P20L376 “*The 7-day recall period of the CGI-Severity, assessed while patients were hospitalized in a controlled, alcohol-fee environment, likely underestimated the severity of alcohol use disorder and may have artificially weakened its association with the AQoLS-Brief. “*

6. I would like to hear more information about excluding patients currently experiencing withdrawal symptoms from the current analysis. What proportion of the sample does this exclude? Does this only include extreme, life-threatening withdrawal symptoms, or even more mild withdrawal symptoms? If it is life-threatening symptoms only then that makes sense for safety purposes. However, exclusion based on mild withdrawal symptoms may bias the sample unnecessarily.

R6. As we required patients to no longer be on benzodiazepines before inclusion, we did not encounter any cases where a patient still experienced withdrawal symptoms and could not be included. To clarify: patients with withdrawal symptoms would have been prescribed benzodiazepines as part of their medically supervised alcohol withdrawal process.

7. I am wondering why other item selection analyses were not used to guide item selection, such as item discrimination and item difficulty. Some rationale as to why these were not used would be helpful.

R7. We already documented item difficulty and discrimination, through ir-ρ correlations. As suggested by the reviewer, we added more analyses to document items discrimination. and difficulty.

Methods: P9L200 “*To further document the relevance of the selection, we calculated additional psychometrics on the item difficulty and discrimination. Item difficulty was assessed by item distribution and the average item score. In order to obtain item difficulty indices (noted* $d$*) comparable across all items in a multiple-item instrument we scaled each average item score to interval [0,1] by accounting for a minimal and maximal possible item score min (0) and max (3) (Martinková and Hladká 2023). Item discrimination was estimated by the upper-lower index, the difference in difficulty between an upper and lower group of respondents based upon their respective dimension scores(Martinková and Hladká 2023) . By a rule of thumb, upper-lower index based upon thirds should not be lower than 0.2, except for very easy or very difficult items (Ebel 1954). Items of medium difficulty with lower upper-lower index are considered suspicious, and their content and wording should be checked*. *.* ”

*Results: P12L276 “Table 3:* Psychometrics of AQoLS items in each dimension and selected items in the AQoLS-Brief

| **Item** | **ir-ρ^a^** | **Upper-lower index** | **Cronbach α^b^** | **Average inter-item correlation** **^c^** | **d^d^** | **Item distribution** |
| --- | --- | --- | --- | --- | --- | --- |
| **Activities** |  |  | 0.87 | 0.40 |  |  |
| 02. Plans | 0.58 [0.49; 0.66] | 0.46 | 0.86 | 0.40 | 0.48 | 15.4 \| 37.9 \| 34.4 \| 12.3 |
| 03. Restricted in places | 0.52 [0.42; 0.61] | 0.51 | 0.86 | 0.41 | 0.42 | 31.3 \| 29.5 \| 22.0 \| 17.2 |
| **04. Physical activities** | **0.74 [0.67; 0.79]** | **0.67** | **0.84** | **0.38** | **0.51** | **21.6 \| 27.3 \| 26.4 \| 24.7** |
| 05. Jobs around the house | 0.67 [0.59; 0.74] | 0.64 | 0.85 | 0.39 | 0.46 | 24.2 \| 34.4 \| 21.1 \| 20.3 |
| 06. Ability to work | 0.60 [0.51; 0.68] | 0.53 | 0.86 | 0.40 | 0.46 | 22.5 \| 31.7 \| 30.0 \| 15.9 |
| 07. Cut myself off | 0.60 [0.51; 0.68] | 0.55 | 0.86 | 0.40 | 0.54 | 16.3 \| 30.4 \| 28.2 \| 25.1 |
| 13. Sex | 0.38 [0.26; 0.49] | 0.39 | 0.87 | 0.44 | 0.42 | 29.1 \| 29.5 \| 26.4 \| 15.0 |
| 15. Household affairs | 0.60 [0.51; 0.68] | 0.59 | 0.86 | 0.40 | 0.48 | 22.9 \| 33.0 \| 21.1 \| 22.9 |
| 25. Appearance | 0.59 [0.50; 0.67] | 0.52 | 0.86 | 0.40 | 0.40 | 30.0 \| 32.6 \| 24.2 \| 13.2 |
| 26. General health | 0.62 [0.53; 0.69] | 0.47 | 0.86 | 0.40 | 0.54 | 11.5 \| 30.8 \| 40.5 \| 17.2 |
| **Relationships** |  |  | 0.83 | 0.45 |  |  |
| 01. Everyday activities | 0.51 [0.41; 0.60] | 0.48 | 0.82 | 0.48 | 0.58 | 09.7 \| 30.4 \| 35.7 \| 24.2 |
| 08. Neglected people | 0.63 [0.54; 0.70] | 0.51 | 0.79 | 0.43 | 0.43 | 23.3 \| 35.7 \| 29.1 \| 11.9 |
| 09. Relationships | 0.69 [0.62; 0.75] | 0.63 | 0.78 | 0.42 | 0.49 | 18.9 \| 34.8 \| 26.9 \| 19.4 |
| 10. Behaved badly | 0.60 [0.51; 0.68] | 0.47 | 0.80 | 0.44 | 0.37 | 27.3 \| 41.9 \| 22.0 \| 08.8 |
| **11. Family** | **0.72 [0.65; 0.78]** | **0.63** | **0.78** | **0.41** | **0.53** | **16.7 \| 31.7 \| 28.2 \| 23.3** |
| 27. Risky situations | 0.45 [0.34; 0.55] | 0.44 | 0.83 | 0.49 | 0.42 | 24.7 \| 36.6 \| 27.3 \| 11.5 |
| **Living conditions** |  |  | 0.75 | 0.43 |  |  |
| 16. Housing situation | 0.46 [0.35; 0.56] | 0.43 | 0.74 | 0.49 | 0.21 | 61.2 \| 22.5 \| 07.5 \| 08.8 |
| 17. Money spent on alcohol | 0.65 [0.57; 0.72] | 0.61 | 0.63 | 0.37 | 0.36 | 36.1 \| 24.7 \| 34.8 \| 04.4 |
| **18. Financial difficulties** | **0.67 [0.59; 0.74]** | **0.56** | **0.62** | **0.36** | **0.30** | **44.9 \| 27.8 \| 20.7 \| 06.6** |
| 24. Appetite | 0.42 [0.31; 0.52] | 0.55 | 0.76 | 0.51 | 0.44 | 26.4 \| 28.6 \| 30.8 \| 14.1 |
| **Negative emotions** |  |  | 0.69 | 0.53 |  |  |
| 22. Worried about my health | 0.53 [0.43; 0.62] | 0.56 | 0.60 |  | 0.59 | 08.4 \| 32.6 \| 32.2 \| 26.9 |
| **23. Worried about my life** | **0.53 [0.43; 0.62]** | **0.51** | **0.47** |  | **0.64** | **04.4 \| 24.7 \| 44.5 \| 26.4** |
| **Self-esteem** |  |  | 0.81 | 0.45 |  |  |
| 12. Trust | 0.59 [0.50; 0.67] | 0.55 | 0.77 | 0.46 | 0.43 | 24.2 \| 36.6 \| 25.1 \| 14.1 |
| 14. Friends | 0.56 [0.46; 0.64] | 0.49 | 0.78 | 0.47 | 0.37 | 27.8 \| 40.5 \| 23.3 \| 08.4 |
| 19. Shame | 0.60 [0.51; 0.68] | 0.53 | 0.76 | 0.45 | 0.70 | 04.0 \| 24.2 \| 30.8 \| 41.0 |
| 20. Contempt | 0.56 [0.46; 0.64] | 0.45 | 0.78 | 0.47 | 0.34 | 32.2 \| 41.4 \| 19.8 \| 06.6 |
| **21. Wasting my life** | **0.64 [0.56; 0.71]** | **0.55** | **0.75** | **0.43** | **0.65** | **05.7 \| 25.6 \| 36.6 \| 32.2** |
| **Control** |  |  | 0.86 | 0.55 |  |  |
| 28. Nothing matters | 0.70 [0.63; 0.76] | 0.60 | 0.82 | 0.54 | 0.42 | 26.4 \| 32.2 \| 30.0 \| 11.5 |
| 29. Alcohol controlled me | 0.71 [0.64; 0.77] | 0.60 | 0.82 | 0.54 | 0.58 | 11.0 \| 28.2 \| 37.0 \| 23.8 |
| **30. Life around alcohol** | **0.77 [0.71; 0.82]** | **0.61** | **0.81** | **0.51** | **0.53** | **14.5 \| 29.5 \| 38.3 \| 17.6** |
| 31. Plan around alcohol | 0.75 [0.69; 0.80] | 0.64 | 0.81 | 0.52 | 0.50 | 19.8 \| 27.3 \| 37.0 \| 15.9 |
| 32. Control of myself | 0.47 [0.36; 0.57] | 0.48 | 0.88 | 0.65 | 0.48 | 19.8 \| 32.2 \| 31.3 \| 16.7 |
| **Sleep** |  |  | 0.87 | 0.77 |  |  |
| 33. Good night's sleep | 0.77 [0.71; 0.82] | 0.77 | 0.78 |  | 0.55 | 19.4 \| 20.7 \| 35.2 \| 24.7 |
| **34. Sleep enough** | **0.77 [0.71; 0.82]** | **0.75** | **0.77** |  | **0.52** | **20.3 \| 26.0 \| 32.2 \| 21.6** |
| **AQoLS-Brief** |  |  | 0.80 | 0.37 |  |  |
| 04. Physical activities | 0.60 [0.51; 0.68] | 0.61 | 0.76 | 0.36 | 0.51 | 21.6 \| 27.3 \| 26.4 \| 24.7 |
| 11. Family | 0.64 [0.56; 0.71] | 0.59 | 0.75 | 0.35 | 0.53 | 16.7 \| 31.7 \| 28.2 \| 23.3 |
| 18. Financial difficulties | 0.37 [0.25; 0.48] | 0.35 | 0.80 | 0.42 | 0.30 | 44.9 \| 27.8 \| 20.7 \| 06.6 |
| 21. Wasting my life | 0.67 [0.59; 0.74] | 0.53 | 0.75 | 0.34 | 0.65 | 05.7 \| 25.6 \| 36.6 \| 32.2 |
| 23. Worried about my life | 0.60 [0.51; 0.68] | 0.42 | 0.77 | 0.36 | 0.64 | 04.4 \| 24.7 \| 44.5 \| 26.4 |
| 30. Life around alcohol | 0.62 [0.53; 0.69] | 0.53 | 0.76 | 0.35 | 0.53 | 14.5 \| 29.5 \| 38.3 \| 17.6 |
| 34. Sleep enough | 0.29 [0.17; 0.40] | 0.36 | 0.82 | 0.44 | 0.52 | 20.3 \| 26.0 \| 32.2 \| 21.6 |
| ^a^Item-rest correlation (ir-ρ), i.e. correlation between the item and the dimension's score formed by all other items. Items in bold are those retained for the dimension in the final AQoLS-Brief respecting the a priori rules of choice. | | | | | | |
| ^b^Cronbach α with all items of the dimension, followed by Cronbach α excluding the particular item of the dimension (called α-drop). The lowest the α-drop for an item is, the more important this item is for this dimension. When the dimension has only 2 items xᵢ and xⱼ then α-drop for item xᵢ is computed as cov(xᵢ, xⱼ)/var(xⱼ) (Revelle 2024) | | | | | | |
| ^c^The average interitem correlations with all items of the dimension, followed by the average interitem correlations excluding the particular item of the dimension (called AIIC-drop). The lowest the AIIC-drop for an item is, the more important this item is for this dimension. It cannot be calculated for dimension with only two items. | | | | | | |
| ^d^Item difficulty: average score of the item divided by its range. | | | | | | |

“*The additional psychometrics on item difficulty, discrimination and internal consistency comforted the choice from the priori rules for all chosen items with a d in the optimal range 0.5-0.8, an upper-lower index >0.2 and an average inter-item correlation within or the closest to the optimal range 0.2-0.4. Only financial difficulty showed a d <0.5 but all other items of this dimension did, so the choice to retain this item was kept.”*

8. This is a minor, but there are so many acronyms it is difficult to follow the manuscript at times. I recommend only using very common acronyms (e.g., AUD, DSM-5) and writing out most other acronyms.

R8. We removed acronyms corresponding to discrete choice experiment and preference-based measure.

9. This is also minor, but please proofread the manuscript for correct grammar. For example, on lines 52-54, it reads: “However, its length could limit its use in clinical trials, where subjective measurements are not often the primary judgment outcome (Luquiens et al. 2012) and there is often numerous and time spending measures.” Instead of “there is often numerous and time spending measures,” I assume the authors mean, “there is often numerous and time intensive measures.” There are several places throughout the manuscript where the wording or grammar is slightly off.

R9. We reformulated this sentence: P4L63 “*numerous time-consuming measures are typically involved. A shorter version would be more convenient for both researchers and participants*”

We proofread the manuscript and reformulated several paragraphs.

Please note that we modified the abstract to reflect the changes to the manuscript:

“*Background:*

*Short versions of health-related quality of life measures are essential for the development of preference-based measures used to obtain the quality-adjustment weights needed to calculate quality-adjusted life years (QALYs) in health economic evaluations. Using data from the randomized TRAIN study, which compared the efficacy of two cognitive training programs in patients with alcohol use disorder (AUD) recently detoxified at five centers in France from 2019 to 2023 (n=227), we report the development and psychometrics of the AQoLS-Brief, a short version of the initial 34-item Alcohol Quality of Life Scale (AQoLS).*

*Methods:*

*Baseline data on, age, sex, AUD severity, drinking characteristics and consequences of alcohol including the AQoLS were collected. One item per dimension of the AQoLS was chosen following predefined rules. Construct validity of the AQoLS-brief was documented: internal consistency was assessed using Cronbach's alpha and external validity was assessed with external drinking and non-drinking variables via univariate analyses, a confirmatory factorial analysis (CFA) and a multivariate linear regression.*

*Results:*

*The 7-item AQoLS-Brief demonstrated good internal consistency (Cronbach alpha= 0.80) and very high correlation with the long version AQoLS (rho=0.95). The CFA confirmed the unidimensionality of the AQoLS-Brief. Severity of AUD, as measured by the number of DSM-5 alcohol criteria and the number of heavy drinking days, and the dimension Sadness of the Affective Neuroscience Personality Scales were the heaviest contributors to the AQoLS-Brief score.*

*Conclusions:*

*The AQoLS-Brief is a short version of the AQoLS with good psychometrics, enhancing its use in clinical practice and research, and representing a significant step toward establishing a relevant and specific preference-based measures for calculating QALYs in AUD populations.*“

Added References:

Davis, Kenneth L, Jaak Panksepp, and Larry Normansell. 2003. 'The affective neuroscience personality scales: Normative data and implications', *Neuropsychoanalysis*, 5: 57-69.

Ebel, Robert L. 1954. 'Procedures for the Analysis of Classroom Tests', *Educational and Psychological Measurement*, 14: 352-64.

Gana, Kamel, and Guillaume Broc. 2018. *Structural Equation Modeling with lavaan*.

Higuchi, S., Y. Moriguchi, and K. H. X. Tan. 2020. 'Psychometric validation of the Japanese version of Alcohol Quality of Life Scale (AQoLS-Japan) in the treatment of patients with alcohol use disorder', *Qual Life Res*, 29: 223-35.

Jöreskog, K.G., D. Sörbom, and SPSS Inc. 1989. *LISREL 7: A Guide to the Program and Applications* (SPSS).

Luquiens, A., M. Reynaud, B. Falissard, and H. J. Aubin. 2012. 'Quality of life among alcohol-dependent patients: How satisfactory are the available instruments? A systematic review', *Drug Alcohol Depend*, 125: 192-202.

Luquiens, A., D. Whalley, S. R. Crawford, P. Laramee, L. Doward, M. Price, N. Hawken, J. Dorey, L. Owens, P. M. Llorca, B. Falissard, and H. J. Aubin. 2015. 'Development of the Alcohol Quality of Life Scale (AQoLS): a new patient-reported outcome measure to assess health-related quality of life in alcohol use disorder', *Qual Life Res*, 24: 1471-81.

Luquiens, A., D. Whalley, P. Laramee, B. Falissard, N. Kostogianni, J. Rehm, J. Manthey, F. Paille, and H. J. Aubin. 2016. 'Validation of a new patient-reported outcome instrument of health-related quality of life specific to patients with alcohol use disorder: the Alcohol Quality of Life Scale (AQoLS)', *Qual Life Res*, 25: 1549-60.

Martinková, P., and A. Hladká. 2023. *Computational Aspects of Psychometric Methods: With R*.

Mokkink, L. B., H. C. W. de Vet, C. A. C. Prinsen, D. L. Patrick, J. Alonso, L. M. Bouter, and C. B. Terwee. 2018. 'COSMIN Risk of Bias checklist for systematic reviews of Patient-Reported Outcome Measures', *Qual Life Res*, 27: 1171-79.

Revelle, W. 2024. "psych: Procedures for Psychological, Psychometric, and Personality Research. ." In *Northwestern University, Evanston, Illinois.* .

Rosseel, Y. 2012. 'lavaan: An R Package for Structural Equation Modeling. ', *Journal of Statistical Software,*, 48: 1-36.

Woicik, P. A., S. H. Stewart, R. O. Pihl, and P. J. Conrod. 2009. 'The Substance Use Risk Profile Scale: a scale measuring traits linked to reinforcement-specific substance use profiles', *Addict Behav*, 34: 1042-55.
