# Supplementary material for: AQoLS-Brief: Development and psychometric properties of a short version of the alcohol quality of life scale
Source: PLoS One. 2025 Apr 29;20(4):e0321365. doi: 10.1371/journal.pone.0321365 (PMC12040188; doi:10.1371/journal.pone.0321365)
Supplement: Attachment — Submitted filename: PLOS ONE review_2_240225.docx [file pone.0321365.s004.docx]

We would like to thank the reviewers for their positive feedback. Below, we provide our detailed response to the last comment.

Reviewer #2: The authors have thoroughly responded to all my critiques. I commend the authors for their comprehensive and well-reasoned responses. The changes made have greatly strengthened the manuscript and increased the manuscripts contribution to the literature. I have one final minor comment to be addressed:

1. In the hypotheses (starting line 215), it would be helpful to specify the hypothesized directions of the associations (positive or negative) in addition to the strength of the correlations.

R1. We added that we expected positive correlations : P9L215: “*We hypothesized a moderate positive correlation with measures of severity (CGI-severity and DSM-5 criteria for AUD), a moderate to low positive correlation with the history of alcohol use disorder (i.e the length since the onset of alcohol use disorder), the drinking outcome (i.e. the number of HDD in the last 4-week drinking period) and dimensions of the psychological scales ANPS and SURPS related to anxiety-depression, i.e. ANPS Sadness and Fear dimensions, and SURPS Anxiety Sensitivity and Hopelessness dimensions.”*
